# Implementing an Integrated Large-Scale Clinical Information System for ISSSTE’s Hospital Network in Mexico

**DOI:** 10.1007/s42399-020-00713-2

**Published:** 2021-01-24

**Authors:** Arturo Cervantes Trejo, Sophie Domenge Treuille, Isaac Castañeda Alcántara

**Affiliations:** 1 Anahuac Institute of Public Health, Carlos Peralta Chair of Public Health, Universidad Anahuac Mexico, Lomas Anáhuac s/n, Edificio CAIDE, sexto piso, 52787 Huixquilucan, CP Mexico; 2 Hospital Regional Pemex Ciudad Madero (Tamaulipas, Mexico) and Anahuac Institute of Public Health, Universidad Anáhuac Mexico, Huixquilucan, Mexico; 3 Anahuac Institute of Public Health, Carlos Peralta Chair of Public Health, Anahuac Institute of Public Health, Universidad Anáhuac Mexico, Huixquilucan, Mexico

**Keywords:** ISSSTE, Mexico, Health informatics, Clinical information system (CIS), Radiology information system (RIS), Clinical data warehouse (CDW), Picture archiving and communication system (PACS), Hospital information system (HIS), Public health

## Abstract

The Institute for Security and Social Services for State Workers (ISSSTE) is a large public provider of health care services that serve around 13.2 million Mexican government workers and their families. To attain process efficiencies, cost reductions, and improvement of the quality of diagnostic and imaging services, ISSSTE was set out in 2019 to create a digital filmless medical image and report management system. A large-scale clinical information system (CIS), including radiology information system (RIS), picture archiving and communication system (PACS), and clinical data warehouse (CDW) components, was implemented at ISSSTE’s network of forty secondary- and tertiary-level public hospitals, applying global HL-7 and Digital Imaging and Communications in Medicine (DICOM) standards. In just 5 months, 40 hospitals had their endoscopy, radiology, and pathology services functionally interconnected within a national CIS and RIS/PACS on secure private local area networks (LANs) and a secure national wide area network (WAN). More than 2 million yearly studies and reports are now in digital form in a CDW, securely stored and always available. Benefits include increased productivity, reduced turnaround times, reduced need for duplicate exams, and reduced costs. Functional IT solutions allow ISSSTE hospitals to leave behind the use of radiographic film and printed medical reports with important cost reductions, as well as social and environmental impacts, leading to direct improvement in the quality of health care services rendered.

**Supplementary Information:**

The online version of this article (10.1007/s42399-020-00713-2) contains supplementary material, which is available to authorized users.

## Introduction

The integration of technology and information systems to improve hospital and clinical performance has widely been practiced around the globe evolving rapidly since the 1960s [1–3]. The USA and other European countries have been pioneers in the use of health informatics to ease the management of hospital information and improve the quality of health care [4]. Since then, many health care systems around the world have reported the challenges and benefits of health informatics, including the implementation of hospital information systems (HISs), electronic medical records (EMRs), radiology information systems (RISs), picture archiving and communication systems (PACSs), clinical data warehouse (CDW), and clinical information systems (CISs) [2, 5–8].

These have since become the norm for successful and efficient medical care, public health services, and clinical practice in the twenty-first century [1, 4]. CIS is considered a sub-system of HIS and is devoted to facilitating and improving the direct management of patients [4]. In contrast to HIS, which is designed to assist administrative and financial management functions of health care systems, the main goal of clinical information systems is to provide information and knowledge to medical providers during the care delivery process.

In Mexico, millions of dollars have been invested during the last decades in information technology for public health care institutions, yet little is known about the results of these investments. In 2003, the National Institute for Rehabilitation, one of the country’s leading public sector institutions, began using medical imaging technology by implementing its high-availability RIS/PACS system [9, 10]. We could not find other literature describing such implementations in Mexico.

Since 1959, the Institute for Social Security and Services for State Workers (ISSSTE) provides health care and social services to all government workers and family members. It is estimated that in 2019, ISSSTE serves over 13.2 million Mexicans [11]. Every year, the ISSSTE provides millions of medical consultations as well as clinical laboratory exams and diagnostic and imaging services. In terms of physical infrastructure, ISSSTE has 1182 medical units divided among primary, secondary, and tertiary care facilities [12]. Of these, 40 are providing secondary- and tertiary-level specialized care.

Between 2010 and 2014, ISSSTE invested more than 800 million dollars in information systems and technological infrastructures [13]. Nevertheless, in 2017, top management at ISSSTE had recognized a disorderly growth of IT infrastructure for hospital management and specifically for the management of clinical images. One of the major and recurring costs of ISSSTE’s radiology services are expenses to processing X-rays. Thus, ISSSTE opts for the use of RIS/PACS technology [14].

RISs are software solutions to automate the workflow of radiology departments and enable scheduling functions and keep single historical files for each patient. Picture archiving and communication systems (PACSs) replace traditional radiological film processes. They are a means for digitally acquiring, storing, transmitting, and displaying diagnostic images. PACS allows for the connection of all imaging services within the radiology department. Nonetheless, few documented experiences allow for full access to diagnostic imaging to all hospital areas or even less so to other hospitals and providers within a health care network [3].

In October 2017, ISSSTE formally started the public purchasing process for a multi-year CIS with RIS/PACS and CDW components to network their regional hospitals (third level of care) and general hospitals (second level of care), equipping them with auxiliary devices for the administration of medical images and management of fundamental diagnostic imaging and endoscopy and pathology studies. The project called for the installation of digitalization, storage, interpretation, image visualization, and patient data management equipment, including the installation of hardware and software components. The terms of the bid required the management of approximately 2 million digital studies and medical reports per year, carried out by the imaging, pathology, and endoscopy departments of their regional and general hospitals throughout 29 states of the country.

Information for each patient was to be treated confidentially, stored safely within the ISSSTE, and available 24 h a day, 365 days a year. Authorized medical or administrative personnel should have unlimited access, regardless of their location. Aside from the information systems and computer programs, ISSSTE required technical assistance to all devices and equipment to be granted by the winning bidder with their own staff 24 h a day, 365 days a year for 36 months beginning in May 2018 and coming to an end in April 2021.

This article presents results from the first 16 months of implementation of ISSSTE’s high-availability CIS linking their network of regional and general hospitals, explaining the main benefits obtained as well as some of the challenges encountered.

## Methods

On December 15, 2017, the Mexican company “TESI de Mexico” was granted the winning bid for the project *“Service of management, storage, and distribution of medical imaging from radiology, endoscopy, and pathology”* for 36 months starting in May 2018 [14]. A two-team effort model was used for the implementation, where the private sector team (TESI) was contracted to fully implement the CIS, RIS/PACS, and CDW solution, and the partner teams at each hospital belong to the public sector, in this case personnel from ISSSTE. The primary advantage of this two-team model is that the RIS/PACS specifications are tailored to each hospital’s environment, yet the responsibility for implementing the CIS and CDW technical solutions and to keep them running relies solely on the private provider [3].

A highly specialized team of 40 engineers led by a supervisor team of 3 experts and project leader was at the core of the implementation. The use of standardized network technology is a prerequisite for digital image communications. Local area network (LAN) was installed in the 40 hospitals using Ethernet 6A cabling. A wide area network (WAN) was also established for these hospitals, allowing communication over large distances and covering 29 states of the country at speeds based on network traffic from 10 to 100 Mbps (megabits per second). Each hospital was equipped with two high capacity redundant servers in a cluster and a national high capacity cluster that stores all the images and medical reports, with a total storage capacity of over 500 TB.

**Fig. 1:**
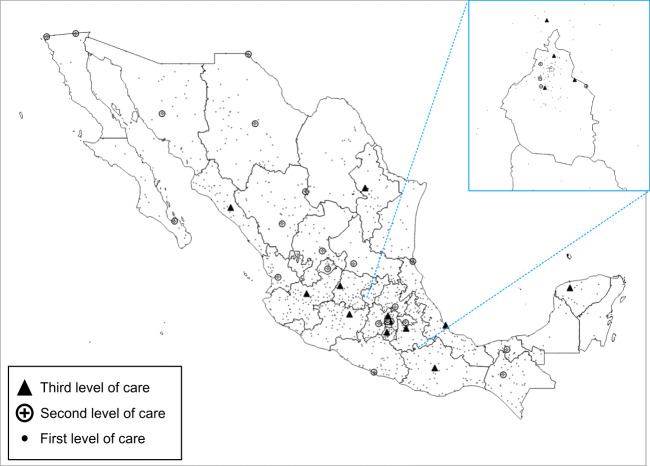
Geographic location of ISSSTE’s physical infrastructure, highlighting the CIS network in 2019

The CIS network includes a national medical center (Centro Médico Nacional Hospital 20 de Noviembre), 14 regional hospitals, and 25 general hospitals, located in 29 states and servicing 11 regional service networks. No primary care clinics or health units were included (Fig. 1). Together, the 40 hospitals have 5027 beds and 202 operating rooms, which represent 100% of ISSSTE’s total hospital capacity. These hospitals are responsible for health care services to more than 740,000 patients each year, perform over 26 million laboratory studies, have most patient admissions, and perform diagnostic procedures, as seen in Table 1 [12, 15].

**Table 1:** Total hospital capacity of ISSSTE compared to the capacity of the CIS network

Category of indicator	Total for ISSSTE hospitals	Total CIS network	Percent of ISSSTE capacity
First level of care	*1058*		
Medical unit in workplace	83	–	
Clinic for family care	478	–	
Family health unit	395	–	
Family health clinic	87	–	
Family health clinic with specialties	15	–	
Second level of care	*108*		
Specialty clinics	12	–	
Hospital clinics	71	–	
General hospitals	25	25	100%
Third level of care	***15***		
Regional hospital/high specialty hospital	14	14	100%
National medical center	1	1	100%
Other health care infrastructures			
Operating rooms	202	202	100%
Outpatient operating rooms	129	–	
Hospital beds	5027	5027	100%
Non-hospitalization beds	1956	–	
Delivery rooms	136	46	34%
Blood banks	50	35	70%
Number of patients			
Emergency room care	717,218	479,722	67%
Hospitalizations	373,773	262,385	70%
Discharges	371,051	260,436	70%
Deaths	15,849	11,844	75%
Number of exams/procedures			
Laboratory	37,607,762	26,736,827	71%
X-ray	1,577,360	1,107,018	70%
Cytology	178,901	140,962	79%
Histopathology	149,168	142,899	96%
Endoscopy (gastro intestinal)	59,688	55,433	93%
Bronchoscopy	3685	3424	93%
Mammography	76,605	58,328	76%
Magnetic resonance imaging (MRI)	28,880	28,875	100%
CT scan (computed tomography)	188,772	179,316	95%
Ultrasound	418,706	276,015	66%

Table 1 Sources:

Subsistema de Información de Equipamiento, Recursos Humanos e Infraestructura para la Atención de la Salud (SINERHIAS), Accessed May 24, 2020. http://www.dgis.salud.gob.mx/contenidos/sinais/subsistema_sinerhias.html

ISSSTE, Anuario Estadístico 2018. Accessed May 222,020. http://www.issste.gob.mx/datosabiertos/anuarios/anuarios2019.html

The implementation process started in January 2018 at “Adolfo López Mateos” Regional Hospital in Mexico City and was completed in May 2018 when all forty hospitals were part of a single network. Before this project, five hospitals already had some types of PACS system installed; nevertheless, these prior systems were discarded. The DICOM images available from existing equipment were accessed and integrated into the new database. The “20 de Noviembre” National Medical Center was chosen as the main IT Hub for the CDW, hardware infrastructure, and ISSSTE’s National Archive of Images and Digital Medical Reports.

CIS, RIS/PACS, and CDW systems were introduced to manage patient scheduling, patient information, imaging technique information, and radiological, pathological, and endoscopy reports, and the whole workflow of each of these services was automated [3, 7]. PACS systems were introduced to meet the demands for more time- and cost-effective image storage and transmission. Both RIS and PACS systems are critical components on which the CIS is based.

Training of local IT technicians and engineers for each hospital took part during the installation process. Additional training was carried out in all hospitals with hands-on workshops where physicians or administrative staff could attend as often as necessary. A total of 2825 h of workshops and training sessions was held for over 4297 medical, administrative, and technical personnel during 12 months. Besides, 24/7 technical support is provided to assist users regarding any problem with the system. This kind of training was chosen as it has shown better results in terms of the ability to use the specialized image and medical reports visualization software [16].

Finally, qualitative interviews regarding the CIS, CDW, and RIS-PACS implementation process and outcomes were conducted by the authors, including systems engineers responsible for the project, as well as doctors, radiologists, pathologists, medical residents, and administrative staff of the imaging, pathology, and endoscopy departments at ISSSTE’s Centro Médico Nacional 20 de Noviembre and at Adolfo López Mateos Hospitals.

### Database Management Technology

The network operates with a unique combination of different software systems: endoscopy (Endox), pathology (Pathox), and radiology (Radox). These systems automate the workflow of each of these services and acquire images and video files to allow the specialist doctor to issue the medical report and send images, videos, and medical reports to the hospital’s repository archive. All studies can be visualized from “TESI.view,” an online viewer that enables complete traceability of patient’s study progress as well as on 2385 viewing stations. All these software components are registered trademarks by “Tesi Group” of Italy and sold by “TESI de Mexico,” the Mexican subsidiary company.

Endox is a web-based system programmed on ASP.Net and Internet Information Services (IIS) and uses a Microsoft SQL Server database. It can be opened from any browser so it does not require installation on computers from where is it accessed. Pathox is a client-server system based on .NET technology, connected to a database that runs on Microsoft SQL Server. Radox consists of 2 systems: RIS (radiology information system) and PACS (picture archiving and communication system). The RIS is a web system programmed on Java and Apache Tomcat using PostgreSQL that stores the radiology service information, including scheduling and basic patient information (age, gender, date of birth). The PACS uses a PostgreSQL database as well, storing all information files related to the different modes of diagnostic images from the patients. Together, these four software components constitute the base of ISSSTE’s clinical information system.

The use of DICOM standards is vital for integrating disparate radiology imaging systems and the equipment that is used in digital radiology, endoscopy, or pathology [10]. This allowed for the connection of most recent imaging devices to a DICOM-compatible archive. It also allowed the connection of older equipment that uses older communication modalities to the PACS image archive through a system developed by TESI that manages any kind of image format.

The data architecture adheres to international standards such as Health Level 7 (HL7) and national guidelines mandated by the Ministry of Health for the National Health System in Mexico. The system is developed in accordance specifically to Technology Guide No. 41 “Picture Archiving and Communication Systems for images (PACS)” and allows for exchange interfaces according to the standards defined in Official Mexican Standard NOM-024-SSA3-2010 [17, 18].

The storage of information in the system is a critical component given that a failure could severely limit the operation of critical hospital areas [10]. The architecture considers security and data redundancy, database backups, and secure storage servers in a UDS (Unified Data Storage) system.

## Results

In 5 months, 40 hospitals of ISSSTE had their endoscopy, radiology, and pathology services functionally interconnected, with operational CIS, RIS/PACS, and CDW systems running on secure private LANs and a secure national WAN. A total of 359 radiology, endoscopy, and pathology image capturing devices were connected to the system (Table 2).

**Table 2:** Number of radiology and endoscopy units that compose the CIS

Name of equipment	Abbreviation	Number
Radiography	Radi	89
(CT scan) computed tomography	CT scan	44
Digital X-ray	X-ray	14
Mammography	Mamm	34
Fluoroscopy	Fluo	14
Endoscopy	Endo	61
Ultrasound	US	79
Angiography	Angio	14
Total		359

A total of 3596 individual units of additional equipment were installed such as label printers, scanners, bar code readers, electronic signature equipment, CD/DVD recording robots, dictation software, microphones, interpretation workstations, microscope image capture equipment, high-resolution monitors, and DICOM interface for analog equipment [14].

Since May 2018, all forty public hospitals in the network are using the CIS, RIS/PACS, and CDW systems as the only diagnostic image viewing options. This means that all data is securely stored in each hospital, in larger regional repositories, and ISSSTE’s National Archive of Images and Digital Medical Reports has been formed. Any study and its interpretation may be accessed at any time by authorized clinicians, including those located outside the hospitals where the images and studies originated.

Table 3 shows that the adoption of the CIS system has been completed and that its regular use has been achieved through the hospitals belonging to the ISSSTE network.

**Table 3:** Comparative of diagnostic procedures done in 2017 and in a 12-month period (September 2018–August 2019)

Category of indicator	Procedures 2017 (12 months)	Procedures CIS network (12 months)	Percent of ISSSTE’s capacity
Number of exams/procedures			
X-ray	1,107,018	1,478,044	134%
Cytology	140,962	102,520	73%
Histopathology	142,899	124,340	87%
Endoscopy	58,857	50,221	85%
Mammography	58,328	62,060	106%
Magnetic resonance imaging	28,875	28,560	99%
CT scan (computed tomography)	179,316	165,030	92%
Ultrasound	276,015	173,530	63%
Positron emission tomography	–	467	
Fluoroscopy	–	26,235	
Angiography	–	45,919	
Secondary image capture	–	18,603	
Hemodynamic studies	–	470	
Total	1,992,270	2,184,305	110%

**Fig. 2:**
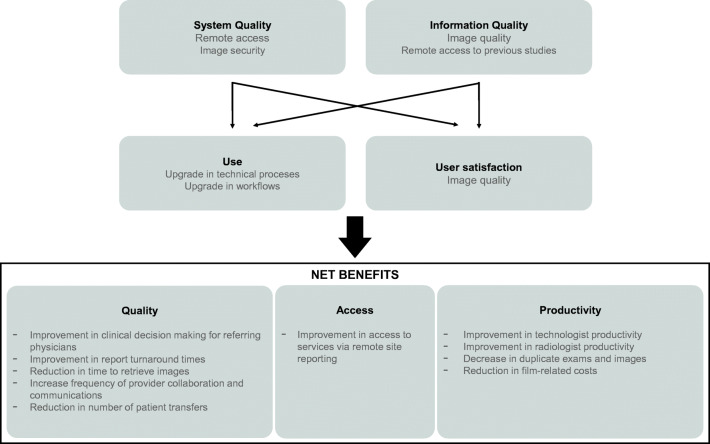
Known benefits of a CIS, CDW, RIS/PACS implementation [19]

As described in Fig. 2, there are multiple benefits derived from the CIS system implementation [2, 3, 5, 6]. Although much of the impact of the CIS and RIS-PACS system remain to be measured, ISSSTE estimates that the largest financial savings are from consumables and operating expenses. Before the system’s implementation, in 2017, an average cost of a radiology exam was $9.43 per patient. In 2019, with the CIS, RIS-PACS, and CDW, this same exam cost only $6.52 per patient. This is equivalent to more than 30% cost savings only for traditional film X-rays. Although yet to be explicitly measured, there is a drastic reduction in usual diagnostic imaging costs [20, 21].

The state-of-the-art CIS, RIS-PACS, and CDW technology has allowed ISSSTE to have a high-speed inter-and intra-hospital communication network that is unique in its history. A single registry of patients has made it possible to have a reliable and secure CDW where patient files are created and contain all imaging, pathology, and endoscopic studies and their interpretations. The contents of the CDW are available 24/7 and accessible from within the hospital network or from remote locations. This has allowed quick and easy access to prior studies, which facilitates comparison and enables peer reviews or second opinions. This has resulted in a strengthened referral and counter-referral processes [21].

Imaging departments at the participating hospitals report greater process efficiency, reflected in the reduction of costs and reduction in environmental impacts of imaging processes at participating hospitals. In terms of operating expense reduction, consumable supplies such as film plates, developing and fixer solutions, and film development equipment have been drastically reduced. Purchase and preventive/corrective maintenance of plate processors have also been minimized. Time optimization has been achieved by the avoidance of repeated image procedures. Internal evaluations conducted by ISSSTE have also documented that early diagnosis and timely treatment have led to reduced hospitalization days and reduced days of paid disability leaves to patients undergoing treatment [21].

From the clinical provider perspective, the viewing interfaces of the PACS system (TesiView) have enabled clinicians with the (previously nonexistent) possibility of processing images with viewing tools that are used to improve image resolution and visualization, which favors the identification of pathologies. This has also been related to a reduction in the number of studies and images taken per patient. Repeat studies have been reduced, and this means that the patient’s exposure to ionizing radiation is also reduced, and time is optimized, with a resulting increase in productivity and efficiency in the imaging departments [21]. Also, physical spaces that were used in hospitals for film development, storage of chemicals, and storage of printed film and files have been reassigned because there is less need for space and physical files have been replaced with digital ones.

The quality and safety of the work carried out in diagnostic imaging departments have improved services provided to patients. The reported benefit of the CIS and CDW includes the availability of digital files for each patient, with secure and reliable information available by remote access. Physicians report more timely and accurate diagnoses, increasing the quality of medical care, in addition to facilitating the work of doctors. Dictation software and equipment, as well as reporting templates, have enabled radiologists to dictate studies that were previously typed, saving time. In a single work station, the radiologist can view all historical images and patient reports. Patient admissions are routinely handled through automated processes using databases and work lists, which has resulted in the avoidance of scheduling errors patients being “lost.” Diagnostic imaging reports can be viewed at any station or hospital computer, and it is no longer necessary to print reports, which has prevented them from getting lost or misplaced.

The benefits observed in pathology departments have also been documented by ISSSTE [20, 21]. Having automation and standardization of processes has resulted in greater efficiency and time optimization in processing pathology studies. Increased security for patient identification has resulted in full traceability during all phases of the pathology department processes. Pathologists are now able to record the findings through dictation software and equipment, or manual transcription, using pre-coded standard phrases and checklists to facilitate the write-up of reports and associate them to ICD-10 coding. These improvements on the management in pathology departments have enabled epidemiologic analysis and the generation of productivity statistics related to TAT, epidemiologic and clinical outcomes, and diagnostic concordance analysis.

Important benefits have also been observed in the endoscopy departments due to the standardization and efficiency in operational processes [21]. The CIS and RIS-PACS components allow the immediate processing of acquired images and video, as well as image archiving in real-time. The video-endoscopes and endoscopic tower equipment are fully compatible and interconnected, regardless of brand. This has enabled an interaction with other HIS and permits the export-import of images directly from the PACS in DICOM formats. This has accomplished the traceability of all studies. Paperless technology has additional environmental benefits by reducing waste and costs related to printing and archiving.

## Discussion

Since their implementation in the 1980s, CIS, CDW, and RIS/PACS systems have revolutionized medical practice at a global scale, presenting multiple advantages compared to conventional archiving and management tools used in hospitals [1, 3–5, 22–28]. Regardless, few hospitals in Mexico are equipped with these systems. Access to clinical and diagnostic imaging information is usually restricted to the hospital units where the patients have done their studies. This causes that patients’ need to repeat imaging or clinical studies that have been done previously or in other hospitals even within the same networks.

Like what has been observed in other global experiences, such as the one in Canada, medical residents and practicing clinicians at ISSSTE have reported improved decision-making, reducing patient transfers, resulting in faster turnaround times, and enabling patient treatment to occur sooner [6, 19, 29, 30]. These secondary outcomes of CIS and CDW remain to be measured. Although ISSSTE is yet to estimate these outcomes, as well as the value of avoided unnecessary patient transfers, these could be in the order of millions of dollars annually, if they are similar to the benefits that have been observed by other CIS and RIS-PACS implementations, as in Canada’s health care system [19].

Other experiences in the world have documented additional benefits of CIS, CDW, and RIS-PACS for patients. The medical literature reports that around 20% of films cannot be found when needed [6], making patients repeat studies for a range of reasons. The loss of films is no longer a problem at ISSSTE’s hospitals. Once an image has been acquired onto the CDW, it cannot be lost, stolen, or misfiled. This presents an enormous advantage to patients as it reduces the need to reschedule appointments, to re-print studies, or to re-expose them to unnecessary radiation or for the time loss due to a missing or lost study.

Given the international experience, over time, it is expected that ISSSTE’s CIS, CDW, and RIS/PACS solutions will result in diverse direct benefits for users, providers, and health care administrators. These benefits include an increase in the timely detection of diseases such as malignant tumors and an improvement in the follow-up of patients. This is because the historical file of patients, including all their X-ray and imaging studies, and their diagnostic interpretations are accessible to physicians in a single system. This has the potential of improving processes for timely detection, diagnosis, treatment, rehabilitation, and palliative care.

If used adequately, the CIS, RIS/PACS, and CDW solutions will allow better administrative controls in the processes of radiology, endoscopy, and pathology departments, reducing costs and providing detailed information on productivity, performance, and quality of health care services. The elimination of costs in supplies formerly used by imaging services, such as film processing and printing, and in processing medical reports from pathology is also expected to generate savings and eliminate the generation of toxic wastes to the environment.

Overall, the CIS is expected to generate improvements in the perception of the quality and satisfaction of the ISSSTE users. This not only improves attention times but also results in a single patient file that facilitates adequate follow-up and better diagnostic processes. Also, by streamlining radiology, endoscopy, and pathology processes, the deferral of medical care and diagnostic studies will be reduced, and so will the average number of hospitalization days. To quantify real benefits obtained by the systems, ISSSTE would benefit from conducting a comprehensive impact and process evaluation to quantitatively and qualitatively document the obtained benefits.

The CIS, RIS/PACS, and CDW systems could also be helpful to ISSSTE for responding to the COVID-19 pandemic. To cope with the growing number of COVID-19 patients, artificial intelligence (AI) applications could easily be implemented by ISSSTE for reducing the burden on clinicians. While a manual read of a CT scan can take up to 15 min, AI can analyze the images in 10 s or less [31]. Thirty-eight of the forty hospitals that implemented this IT solution are operating as COVID-19 centers, and they are currently waging a tireless battle against the disease.

In other parts of the world, RIS/PACS infrastructures are being used in support of the diagnosis and clinical treatment of patients with this disease. State-of-the-art AI applications have recently been developed, using algorithms trained to detect patients with COVID-19 disease from computed tomography images and chest radiographs. In addition to facilitating diagnosis with high precision, in the absence of confirmatory laboratory tests or the case of false negatives, the ISSSTE system could be used for a rapid implementation of AI add-ons, for support in diagnosis and monitoring of COVID cases.

AI could also be used to address ISSSTE’s existing backlog of other imaging department study interpretations. From January to April 2020, an average of 260 CT scans and 5000 chest X-ray studies were performed weekly. These studies are interpreted manually, and, due to increased workload, they are not always interpreted by radiologists, resulting in large backlogs. For example, of 77,060 mammogram studies carried out during 2019, there is an interpretation lag of 17.34%, leaving 13,351 studies uninterpreted in that period. Strategies to reduce the existing lags in the diagnostic interpretations imaging studies could be supported by the CIS, CDW, and RIS/PACS systems, coupled with existing AI solutions.

ISSSTE achieved the creation of a unique filmless technological platform, which could serve as an example for other public and private health care institutions to follow. The speed of this nationwide implementation for ISSSTE in Mexico stands in contrast to the Canadian Diagnostic Imaging (DI) program, which was launched in 2003 and by 2018 had accomplished nearly 100% of “filmless coverage” of all public acute care hospitals across Canada [19]. For this effort, Health Canada invested over 368 million dollars in 39 diagnostic imaging PACS and RIS projects, spanning every province and territory during a 15-year timeframe. For the Mexican effort, a 5-month implementation was possible with a multi-year public investment worth around 13.5 million USD per year.

## Conclusion

Various studies have shown the advantage of CIS, CDW, and RIS/PACS systems for increasing productivity, improving the quality of patient service, and reducing overall hospital costs [5–8, 19, 29, 30]. The efficiency of these systems is due mainly through the elimination of manually intensive tasks associated with the production of film and distribution to physicians, as well as from reductions in time lost and related savings for hospitals and patients [5, 22, 23, 32].

This innovative CIS, RIS/PACS, and CDW infrastructure at ISSSTE’s secondary- and tertiary-level hospitals is a first of its kind for the country and has generated enhanced collaboration between specialists from different medical units across the network of health providers. The CIS solution that integrates all imaging services at these specialty hospitals effectively responds to the need for health care providers to have immediate access to diagnostic images and their interpretations. This need is common at most public hospitals in Mexico. The improved IT infrastructure, consisting of CIS, RIS/PACS, and CDW interfaces, allows for improvement in health care delivery, without increasing service delivery costs.

Also, the clinical information stored in the CDW system allows ISSSTE to access all data immediately using business intelligence (BI) tools. This represents a great leap forward for a public institution that is now able to perform analysis of Big Data and convert information to knowledge. Health care providers benefit from understanding the precise characteristics of the high volumes of patients, the exams, the productivity of equipment, the productivity of personnel, and the timeliness of services (days required to get an appointment, to have access to results and turnaround time) [33]. The availability of this Big Data on the CIS and CDW platform, operating in Mexico’s second-largest public health sector provider, which serves 10% of Mexico’s population, sets an important precedent to follow for other public and private health care providers in the country and the region to reduce costs and improve the quality of care.

The development of this first of its kind clinical information system, RIS/PACS, and CDW network is the first step toward the integration of efficient HIS in Mexico, using a “bottom-up” component-based approach for implementation. Other software systems for managing clinical services such as blood banks, laboratories, and hemodynamic services are being piloted to further integrate clinical information to the CIS network. Major challenges remain and much effort will be needed to integrate other HISs used at ISSSTE to the CIS, RIS/PACS, and CDW platforms. This work is pending and would allow ISSSTE to achieve complete interoperability among separate hospital and clinical management technologies.

Finally, given the important financial, technological, and human resources invested in this CIS, RIS/PACS, and CDW implementation, a comprehensive impact evaluation is recommended for ISSSTE, which would help ascertain and document the cost-effectiveness and the diverse process and outcome improvements brought forth (to the hospitals, providers, and patients) by this innovative technological solution.

## Supplementary information

ESM 1 (PDF 1.99 mb)

## Data Availability

Given the confidentiality of clinical records and patient information, the RIS-PACS clinical data warehouse and business intelligence tools may be made available for the consultation of the SN-CCM editors or other interested relevant parties, upon written request and authorization by ISSSTE authorities and TESI de Mexico, SA de CV. The data for ISSSTE’s health units is available from the SINERHIAS system (Subsistema de Información de Equipamiento, Recursos Humanos e Infraestructura para la Atención de la Salud): http://www.dgis.salud.gob.mx/contenidos/sinais/subsistema_sinerhias.html
