# Supplementary material for: Implementing an Integrated Large-Scale Clinical Information System for ISSSTE’s Hospital Network in Mexico
Source: SN Compr Clin Med. 2021 Jan 24;3(2):444–53. doi: 10.1007/s42399-020-00713-2 (PMC7826294; doi:10.1007/s42399-020-00713-2)
Supplement: Supplementary file 1 — (PDF 1.99 mb) [file 42399_2020_713_MOESM1_ESM.pdf]

Apéndice 25

AVISO DE PRIVACIDAD INTEGRAL

El Instituto de Seguridad y Servicios Sociales de los Trabajadores del Estado como parte del Gobierno Federal protegerá tus datos personales proporcionados a través de la Jefatura de Servicios de Adquisición. Por esta razón, se da a conocer a sus usuarios el siguiente aviso de privacidad integral, basado en la normatividad vigente aplicable a la protección de datos personales.

Objeto.

Informar a los usuarios de la existencia y características principales del tratamiento al que serán sometidos sus datos personales y protección de los mismos, que sean recolectados por Jefatura de Servicios de Adquisición, a fin de que pueda tomar decisiones informadas al respecto.

La denominación del Responsable.
La Jefatura de Servicios de Adquisición en la Subdirección de Recursos Materiales y Servicios.

El domicilio del Responsable.
Av. San Fernando #547, Edificio "F" Primer Piso, Colonia Barrio de San Fernando, C.P. 14070, Tlalpan, Ciudad de México.

Datos personales que serán sometidos a tratamiento (identificando aquéllos que son sensibles).
Los datos que se recaban a través de la Jefatura de Servicios de Adquisición, no se consideran como datos sensibles de conformidad con las disposiciones aplicables, y consisten en datos de identificación y localización.

Cabe aclarar que respecto de los datos personales que proporcione referentes a terceras personas, se presume que usted ha obtenido el consentimiento del titular de que se trate para efectuar dicha entrega.

Tratándose de datos personales de menores de edad y personas en estado de interdicción o incapacidad declarada, se presume que usted cuenta con la representación legal prevista en la legislación civil que le resulte aplicable.

Finalidades del tratamiento para las cuales se obtiene los datos personales (distinguiendo aquellas que requieran el consentimiento del titular).

Los datos personales podrán ser recabados por la Jefatura de Servicios de Adquisición, podrán ser tratados sin consentimiento del titular, siempre en respeto a sus derechos; teniendo como supuestos de excepción a los principios que rigen el tratamiento de datos, la seguridad nacional, disposiciones de orden público, seguridad y salud pública o para proteger los derechos de terceros, según lo establece el segundo párrafo del artículo 16, de la Constitución Política de los Estados Unidos Mexicanos.

Fundamento legal para llevar a cabo el tratamiento.

La Jefatura de Servicios de Adquisición, hace de tu conocimiento que el fundamento para tratar tus datos personales en el Instituto de Seguridad y Servicios Sociales de los Trabajadores del Estado como parte del Gobierno Federal, se encuentra en los artículos 6° base A y 16 segundo párrafo de la Constitución Política de los Estados Unidos Mexicano; 3°, fracción XXXIII, 4°, 16, 17, 18 y 19 de la Ley General de Protección de Datos Personales en Posesión de Sujetos Obligados; 1° y 37, fracción XXIX de la Ley Orgánica de la Administración Pública Federal.

También se informa al titular que no se realizarán transferencias de datos personales que requieran de su consentimiento.

Mecanismos, medios y procedimientos disponibles para ejercer los derechos ARCO.

La Jefatura de Servicios de Adquisición permite al titular ejercer los derechos de acceso, rectificación, cancelación y oposición de datos personales de conformidad con el artículo 16 párrafo segundo de la Constitución Política de los Estados Unidos Mexicanos, así como Título Tercero, Capítulos I y II de la Ley General de Protección de Datos Personales en Posesión de Sujetos Obligados.

Para ejercer estos derechos el titular podrá acudir a Av. San Fernando #547, Edificio "F" Primer Piso, Colonia Barrio de San Fernando, C.P. 14070, Tlalpan, Ciudad de México.

El domicilio y responsable de la Unidad de Transparencia.
Av. Jesús García Corona No. 140, Colonia Buenavista, Delegación Cuauhtémoc, Ciudad de México, C.P. 06350
Horario de atención de la Unidad de Transparencia: de 9:00 a 18:00
Teléfono y extensión: 51409617 ext. 13394 y 13322
Correo electrónico oficial: ventanillaenlace@issste.gob.mx
Nombre del responsable de la atención y operación de la Unidad: Mtra. Benita Hernández Cerón Titular de la Unidad de Transparencia

Cambios y actualizaciones al Aviso de Privacidad.
El presente Aviso de Privacidad puede cambiar o actualizarse periódicamente; por lo que te pedimos lo revises constantemente, para que puedas estar al tanto de la última versión que rige el tratamiento de tus datos personales. No obstante lo anterior, el Aviso de Privacidad siempre deberá observar las disposiciones jurídicas aplicables.

Acepto y conozco el Aviso de Privacidad

Nombre y firma del representante del Licitante
